# Bovine TB in New Zealand – journey from epidemic towards eradication

**DOI:** 10.1186/s13620-023-00248-7

**Published:** 2023-08-30

**Authors:** Jane Sinclair, Dallas New, Mark Neill

**Affiliations:** OSPRI, 15 Willeston St, Wellington, 6011 New Zealand

**Keywords:** Bovine tuberculosis, New Zealand, OSPRI, Brushtail possum, Eradication

## Abstract

Bovine tuberculosis (TB), caused by *Mycobacterium bovis*, has a unique and complex ecology in New Zealand. Unlike elsewhere in the world, the disease is maintained in Australian brushtail possums (*Trichosurus vulpecula*) and so they are considered a vector for disease transmission in New Zealand. Possums were initially introduced to the country in the 1800’s to establish a fur industry but later becoming a recognized pest to native New Zealand flora and fauna. The TB programme in New Zealand (TBFree NZ Ltd) is managed by a not-for-profit limited company partnership between primary industries and government (OSPRI – Operational Solutions for Primary Industries) that uses the basic tenets of disease management, movement control and vector control to eliminate TB in farmed cattle and deer. Evidence of resounding success in the TB control programme resulted in the 2016 decision to pursue full biological eradication of disease from the country by 2055, with the interim objectives of TB freedom in livestock herds by 2026 and TB freedom in possums by 2040. The programme has progressed from an all-time high of 1698 infected herds in 1995 to the lowest recorded point prevalence of 18 infected herds in May 2022. Enhancements that have contributed to the success of the programme include testing with gamma-interferon release assay (Bovigam™) of animals in infected herds that are negative to the skin test (parallel interpretation), culturing pooled lymph nodes from animals without visible lesions, increased testing of herds post-clearance and introduction of post-movement testing of high-risk animals.

## Background

### History of TB in New Zealand and its complex ecology

Bovine tuberculosis (TB) was introduced into New Zealand with the arrival of settlers from Europe, bringing cattle with them in the late 1800’s [1]. By the 1940’s TB had become widespread in the dairy industry and a voluntary testing scheme was introduced followed by a government run programme in the 1960’s. However, the usual “test and slaughter” policy that had proven successful in other countries and in some areas of New Zealand did not prevent recurrent TB infection in livestock in some geographic areas [2]. Other mechanisms of spread and persistence were investigated and in 1969 the first TB infected possum was found. Australian brushtail possums (*Trichosurus vulpecula*) had been introduced into New Zealand beginning in the 1830’s to establish a fur industry, and the abundant availability of food sources and favourable habitat allowed them to reach much higher densities than found in their native Australia [1]. Experiments were conducted that identified possums as a source of disease transmission to livestock.

Over the subsequent twenty years the areas where TB-infected possums were found expanded across New Zealand. The detection of TB infection in livestock peaked in 1995 with a point prevalence of 1698 infected herds. Possums had become well known as a pest in New Zealand due to destruction of forest by over-browsing and impact on native birds by eating eggs and chicks. As an introduced pest species, there have been minimal public concerns with the widespread control of possum populations and in 2016 the New Zealand government included the species in their Predator Free 2050 Campaign.

### How has it been managed?

By the mid 1990’s a centrally run scheme that was backed by legislation was established to address the epidemic of bovine TB. *Mycobacterium bovis* was declared an “unwanted organism” under the Biosecurity Act 1993 [2]. The Animal Health Board (AHB) was established as the non-governmental management agency tasked with controlling the disease through a series of National Pest Management Plans (NPMP) enabled by the Act.

Three basic tenets of the control measures were adopted (and continue to be utilized today):

(i) Disease Management – Cattle and deer surveillance through on-farm skin testing using the caudal fold test (CFT) in cattle and the mid-cervical test (MCT) in deer with slaughter of reactors to remove diseased animals. The age eligibility and frequency of testing is determined by the local risk posed by infected possums and varies across the country. Infected herds are subjected to intense testing and require at least two clear whole herd tests. The final clearance test must be at least six months after the first clear test before movement restrictions are lifted [3]. In addition to this, animals slaughtered for human consumption in New Zealand are required to have a post-mortem inspection which includes identification of TB-like granulomas.
(ii) Movement Control – Movement of cattle and deer is controlled at both area level and herd level (enabled by the Biosecurity Act 1993). Movement Control Areas (MCA) restrict movement of cattle and deer from areas deemed to be at high risk of disease from possums [3]. This involves pre-movement testing within the preceding 60 days of any movement that is not directly to slaughter. Infected herds have their stock movements controlled through the imposition of a Restricted Place Notice issued for the property.
(iii) Vector control – Reducing the density of possum populations, which decreases the transmission of disease between possums and causes the disease to eventually die out in that species. Requiring a long-term systematic landscape-based approach to possum control, with the goal to decrease the number of areas where TB persists in wildlife. Areas where there is a known risk from TB infection in possums are called Vector Risk Areas (VRA). Controlling possum populations consistently decreases the prevalence of disease in livestock and other wildlife species.

In June 2012 New Zealand introduced a National Animal Identification and Tracing (NAIT) system and in July 2013 NAIT was merged with the AHB to obtain some efficiency gains as both programmes were financed by the same organisations. OSPRI (Operational Solutions for Primary industries) was created as a not-for-profit limited company made up of both TBfree NZ Ltd and NAIT Ltd.

Good progress was made controlling bovine TB since the establishment of the first NPMP (1998). By 2011 the objective changed from controlling disease to the investigation of the feasibility of eradication. A system for measuring the confidence that disease had been eliminated from a specific area was developed. The “Proof of Freedom” process utilises spatially explicit, stochastic probability modelling based on disease history, possum habitat across a landscape, possum control history and surveillance activities undertaken [4]. The outputs are compiled with the epidemiological information available, and the case is reviewed by an independent panel for each VRA of interest. Once an area has been declared free of TB it enables funds to be used on another VRA to continue the process, rolling across the landscape.

Confidence in the progress being made resulted in the latest NPMP (2016) having a full eradication goal with three staged milestones:

(i) TB Freedom in livestock herds by 2026, and
(ii) TB Freedom in possums by 2040, and
(iii) Biological eradication of *Mycobacterium bovis* by 2055

## Progress to date

The TB control programme is currently funded by a combination of industry (60% from dairy, beef and deer) and government (40%) contributions and farmer-run regional committees act as testing grounds for policy changes and communication networks.

One of the biggest challenges OSPRI faces has been to balance three competing priorities to the available budget. These include:

1. Cost to deliver the programme
2. Risk within the programme
3. Timeliness of outcomes

Availability of funding is not the only limitation to conducting required control activities. Other reasons include gaining access to privately owned land, considerations of impact of control on endangered native species (kea, a native parrot) and hunting interest groups. Delays to required work can be costly to the programme and introduce additional risk to livestock herds. There have been several areas where delayed control has resulted in clusters of infected herds. Clusters of infection in livestock create an additional risk of spread due to movement, as New Zealand’s cattle move extensively across the country. For example, TB-infected livestock were found in a vector-free area from the movement of dairy service bulls who had originated from an infected herd cluster on the North Island.

**Fig. 1:**
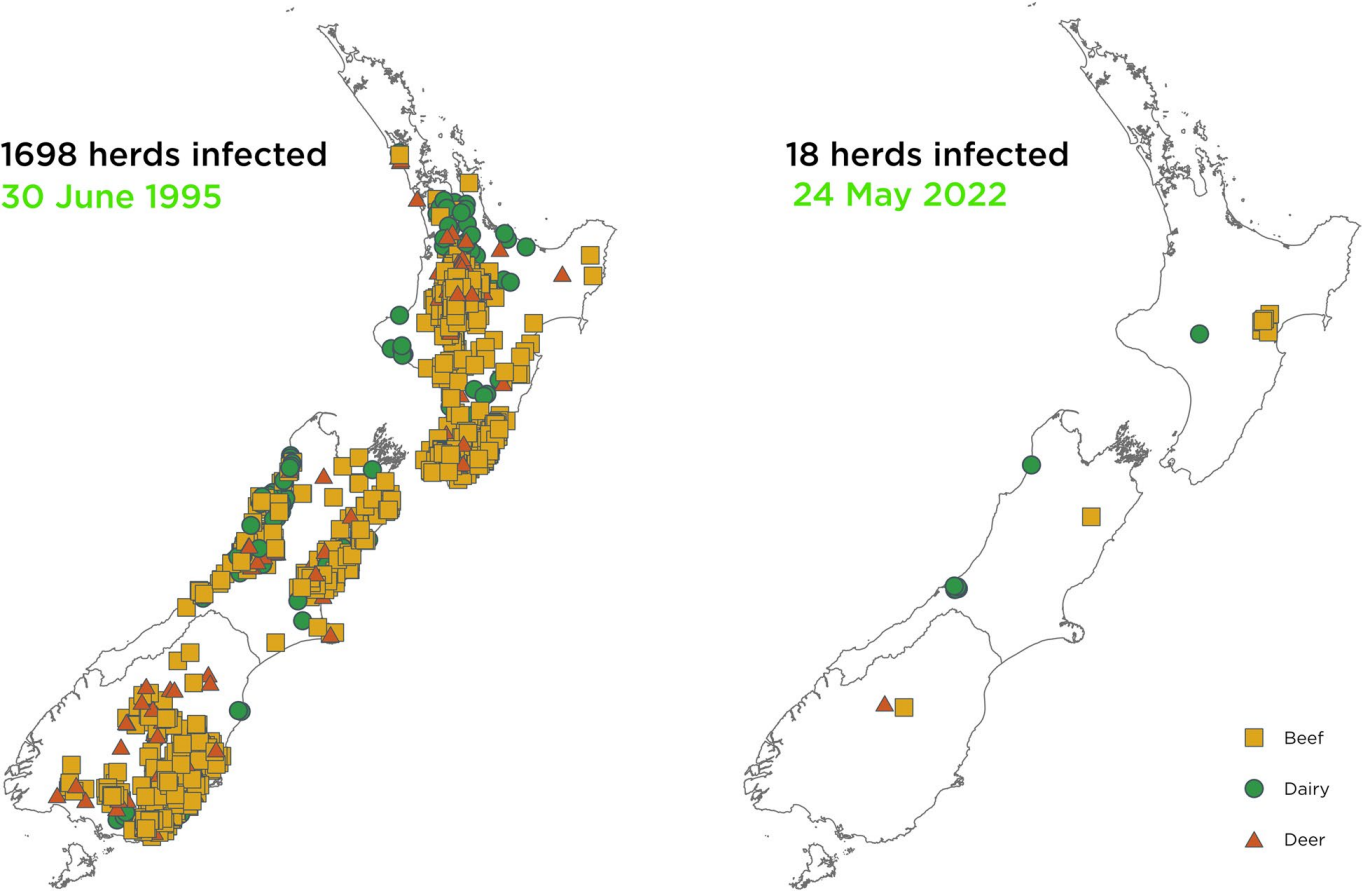
Infected herds in New Zealand from the peak in 1995 to the lowest recorded level in May 2022 (orange squares beef herds, green dots dairy herds and red triangles deer herds)

Despite these challenges, the programme still managed to achieve the lowest point prevalence of TB recorded in New Zealand of 18 infected herds on the 24 May 2022 (Fig. 1).

New Zealand has reduced the area classified as Vector Risk Area (VRA) where infection in possums exists, from 10 million hectares to just over 6 million hectares.

### Programme enhancements

The success of the TB eradication programme has been achieved through application of the programme principles and increasing scrutiny of our surveillance and infected herd management. Previously infected herds are at greater risk of being identified with infection again, than other herds. One reason for this, other than the location of the property, is the fact that some infected animals are not detected at every TB skin or blood test (i.e. none of the TB diagnostic tests have 100% sensitivity). Mitigations adopted in the programme include:

- Regular use of Bovigam™ blood testing (Interferon gamma) on CFT negative animals (parallel interpretation) in infected herds, where positive animals are slaughtered for a disease determination. Only where no confirmed TB cases are found is the test considered a clear test.
  - ◦ Since 2011 this test has been increasingly applied to breeding dairy cattle as they have the greatest risk of delayed detection due to longer productive lifespan than meat production animals.
- Culturing pooled lymph nodes of TB test reactors where no visible lesions were identified at post-mortem examination
  - ◦ This has been applied where there has been a high suspicion of disease in reactors without the expected granuloma lesions being detected at slaughter. Two pools of lymph nodes (both retropharyngeal in one pool and thoracic lymph nodes in the other) are collected and cultured. This approach identified the index TB case in 18% of infected herds in two recent clusters of infection and identified an additional 31 TB-infected animals in those clusters.
- Increased testing of herds post-clearance carried out six monthly. With an additional blood test in CFT negative animals (parallel interpretation) carried out on dairy herds three years after movement restrictions have been lifted.
- Post-movement testing of stock that have moved from previously infected herds

At any stage during the testing process for a herd that an animal is found with TB, then the herd will be deemed infected and movement restrictions applied. If the herd was already infected, then the whole process of disease clearance will commence again (i.e. requiring the two clear whole herd tests six months apart).

## Conclusions

### What does the future hold?

New Zealand continues to progress towards bovine TB eradication, but several challenges have been identified that need to be acknowledged and addressed.

The loss of knowledge and expertise within all aspects of the programme is inevitable when a programme runs over many decades. Retirement of long serving personnel could be better mitigated by good succession planning.

Maintaining farmer awareness and commitment to funding of the programme are vital. An ever-increasing proportion of the farming population has never experienced TB and yet everyone is paying for the programme. Ensuring that we protect the enormous investment that farmers have historically made and continue to make, is essential to achieve a sustainable result. Since the last TB Plan review in 2016, farmers have been inundated with many competing priorities such as climate change, clean water legislation requirements, *Mycoplasma bovis* eradication programme and increasing compliance costs*.*

The next phase of the eradication programme will involve the development of additional methodologies to prove disease absence. The tried and proven methods will continue to be used in addition to new methods and innovation as it becomes available. Work will continue until sufficient evidence is obtained to provide confidence that we have reached our destination.

## Data Availability

Data sharing is not applicable to this article as no datasets were generated or analysed during the current study.

## Abbreviations

AHB: Animal Health Board
CFT: Caudal fold test
MCA: Movement control area
MCT: Mid cervical test
NAIT: National Animal Identification and Tracing
NPMP: National Pest Management Plan
OSPRI: Operational Solutions for Primary Industries
TB: Bovine tuberculosis
VRA: Vector risk area
